# Glutamate dehydrogenase and glutamine synthetase are regulated in response to nitrogen availability in *Myocbacterium smegmatis*

**DOI:** 10.1186/1471-2180-10-138

**Published:** 2010-05-11

**Authors:** Catriona J Harper, Don Hayward, Martin Kidd, Ian Wiid, Paul van Helden

**Affiliations:** 1 DST/NRF Centre of Excellence for Biomedical TB Research, Department of Molecular Biology and Human Genetics, Faculty of Health Sciences, University of Stellenbosch, P.O.Box 19063, Tygerberg, South Africa; 2 Centre for Statistical Consultation, Dept of Statistics and Actuarial Sciences University of Stellenbosch Private Bag X1, Matieland, South Africa

## Abstract

**Background:**

The assimilation of nitrogen is an essential process in all prokaryotes, yet a relatively limited amount of information is available on nitrogen metabolism in the mycobacteria. The physiological role and pathogenic properties of glutamine synthetase (GS) have been extensively investigated in *Mycobacterium tuberculosis*. However, little is known about this enzyme in other mycobacterial species, or the role of an additional nitrogen assimilatory pathway via glutamate dehydrogenase (GDH), in the mycobacteria as a whole. We investigated specific enzyme activity and transcription of GS and as well as both possible isoforms of GDH (NAD^+^- and NADP^+^-specific GDH) under varying conditions of nitrogen availability in *Mycobacterium smegmatis *as a model for the mycobacteria.

**Results:**

It was found that the specific activity of the aminating NADP^+^-GDH reaction and the deaminating NAD^+^-GDH reaction did not change appreciably in response to nitrogen availability. However, GS activity as well as the deaminating NADP^+^-GDH and aminating NAD^+^-GDH reactions were indeed significantly altered in response to exogenous nitrogen concentrations. Transcription of genes encoding for GS and the GDH isoforms were also found to be regulated under our experimental conditions.

**Conclusions:**

The physiological role and regulation of GS in *M. smegmatis *was similar to that which has been described for other mycobacteria, however, in our study the regulation of both NADP^+^- and NAD^+^-GDH specific activity in *M. smegmatis *appeared to be different to that of other Actinomycetales. It was found that NAD^+^-GDH played an important role in nitrogen assimilation rather than glutamate catabolism as was previously thought, and is it's activity appeared to be regulated in response to nitrogen availability. Transcription of the genes encoding for NAD^+^-GDH enzymes seem to be regulated in *M. smegmatis *under the conditions tested and may contribute to the changes in enzyme activity observed, however, our results indicate that an additional regulatory mechanism may be involved. NADP^+^-GDH seemed to be involved in nitrogen assimilation due to a constitutive aminating activity. The deaminating reaction, however was observed to change in response to varying ammonium concentrations which suggests that NADP^+^-GDH is also regulated in response to nitrogen availability. The regulation of NADP^+^-GDH activity was not reflected at the level of gene transcription thereby implicating post-transcriptional modification as a regulatory mechanism in response to nitrogen availability.

## Background

**Figure 1:**
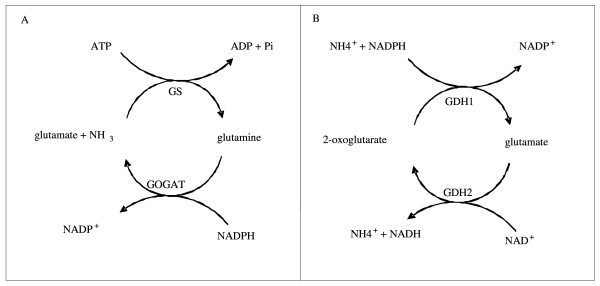
**Assimilation of nitrogen by (A) GS and GOGAT; (B) NADP^+ ^- dependant-glutamate dehydrogenase (GDH1) and NAD^+^-dependant glutamate dehydrogenase (GDH2)**.

Nitrogen is incorporated into glutamate and glutamine which form the major biosynthetic donors for all other nitrogen containing components in a cell. Glutamine is a source of nitrogen for the synthesis of purines, pyrimidines, a number of amino acids, glucosamine and ρ-benzoate, whereas glutamate provides nitrogen for most transaminases [1] and is responsible for 85% of nitrogenous compounds in a cell [2]. In most prokaryotes, there are two major routes for ammonium assimilation. The glutamine synthetase (GS) and glutamate synthase (GOGAT) cyclic mechanism is largely active when exogenous nitrogen concentrations are limiting, due to the high affinity of GS for ammonium. This pathway utilizes approximately 15% of the cell's ATP requirement [1] for the production of glutamine and its activity is, therefore, strictly regulated at both transcriptional and post-translational levels in order to prevent energy wastage (see Figure 1A).

Under conditions of nitrogen excess, glutamine synthetase activity is reduced via adenylylation by the adenylyltransferase GlnE [3,4] and under these conditions, the low ammonium affinity glutamate dehydrogenase (GDH) pathway plays a major assimilatory role with a comparatively low associated energy cost [5]. GDH enzymes catalyse the reversible amination of α-ketoglutarate to form glutamate (see Figure 1B) with concomitant reduction of NAD(P)H. They also serve as metabolic branch enzymes as the GDH enzymes are involved in anapleurotic processes which regulate the flux of intermediates such as α-ketoglutarate between the Krebs cycle and nitrogen metabolism [6]. The GDH enzymes identified in prokaryotes usually function with either NADP^+ ^(EC 1.4.1.4) or NAD^+ ^(EC 1.4.1.2) as co-factors whilst in higher eukaryotes the enzymes have dual co-factor specificity (EC 1.4.1.3). NADP^+^-specific enzymes are normally involved in the assimilation of nitrogen via amination of α-ketoglutarate [7] and may be transcriptionally regulated by a variety of growth conditions, including carbon and nitrogen limitation [8-11]. In contrast, NAD^+^-specific GDH enzymes are thought to be largely involved in glutamate catabolism (deamination) [12-14] and do not appear to be regulated in response to ammonium limitation [15,16]. GDH enzymes described to date are oligomeric structures and can be grouped into three subgroups according to subunit composition. Many NADP^+^- and NAD^+^-GDH enzymes from a number of organisms are hexameric structures made up of subunits that are approximately 50 kDa in size [6]. The second GDH class comprise NAD^+^-specific GDH enzymes with tetrameric structures whose subunits have a molecular mass of approximately 115 kDa [17]. Recently, a third class of oligomeric NAD^+^-specific GDH enzymes was defined whose subunits are approximately 180 kDa in size [18-20].

Information regarding nitrogen metabolism and its regulation in the mycobacteria is relatively limited. Glutamine synthetase (encoded by *glnA1*) has traditionally formed an isolated focal point of study with regard to nitrogen metabolism in the mycobacteria as it has been associated with *Mycobacterium tuberculosis *virulence and pathogenicity [21,22]. It has previously been demonstrated that GS from pathogenic mycobacterial species such as *M. tuberculosis *and *M. bovis *is exported, [23] yet the reasons for this phenomenon and the mechanism of export remain obscure [24]. It has been speculated that extracellular GS may play a role in the production of poly-L-glutamine-glutamate [25], a polymer found only in pathogenic mycobacterial cell walls, and/or that extracellular GS activity may modulate phagosome pH and thereby prevent phagasome-lysosome fusion [23,24]. Comparatively little is known about GS in other mycobacterial species, such as *Mycobacterium smegmatis*, or GDH in the mycobacteria as a whole. The *M. smegmatis *genome encodes for a variety of putative glutamine synthetase enzymes which encode for each of the four possible classes of GS proteins [26], many of which serve unknown functions. Of these homologs, *msmeg_4290 *has the greatest amino acid identity to *glnA1 *in *M. tuberculosis*, which encodes for a GS type 1 ammonium assimilatory enzyme [27]. The *M. smegmatis *GS seems different to *M. tuberculosis *GS in that it does not appear to be expressed to such a high level, nor does it appear to be exported to the extracellular milieu [23,24].

The *M. smegmatis *genome also encodes for an NADP^+^-GDH (*msmeg_5442*) which was isolated by Sarada *et al*. [28]; an L_180 class NAD^+^-GDH (*msmeg_4699*) [29] as well a second putative NAD^+^-GDH enzyme (*msmeg_6272*). In contrast, the *M. tuberculosis *genome only encodes for a single putative NAD^+^-specific GDH (*Rv2476c*) whose activity was detected in culture filtrates by Ahmad *et al *[30]. The enzyme shares a 71% amino acid identity with MSMEG_4699 and may also belong to the L_180 class of NAD^+^-GDH [18,29].

NAD^+^-specific glutamate dehydrogenases belonging to the L_180 class have been characterised in four organisms to date, namely *Streptomyces clavuligerus *[18], *Pseudomonas aeruginosa*[20], *Psychrobacter *sp. TAD1 [31] and *Janthinobacterium lividum *[19], however little functional work has been done on these enzymes. It has very recently been found that the NAD^+^-GDH (MSMEG_4699) isolated from *M. smegmatis *may belong to this class and that it's activity is affected by the binding of a small protein, GarA. This small protein is highly conserved amongst the actinomycetes and was given the name glycogen accumulation regulator (GarA) due to its observed effects on glycogen metabolism in *Mycobacterium smegmatis *[32], however it's precise function remained unclear at the time. GarA has a fork-head associated (FHA) domain which is able to mediate protein-protein interactions as well as a highly conserved N-terminal phosphorylation motif in which a single threonine residue may be phosphorylated by either serine/threonine kinase B (PknB) [33] or serine/threonine kinase G (PknG) [29] thereby presumably playing a role in phosphorylation-dependant regulation mechanisms [34]. It has been shown that Odh1 (the GarA ortholog in *C. glutamicum*; 75% amino acid identity) is able to bind 2-oxoglutarate dehydrogenase, a key TCA cycle enzyme, and cause a reduction in it's activity. This inhibition of enzyme activity was removed by phosphorylation of Odh1 by PknG [35]. A similar phenomenon has been observed in *M. smegmatis *with regards to the modulation of NAD^+^-GDH by GarA. Native or unphosphorylated GarA has been shown to be able to interact with NAD^+^-GDH causing a reduction in NAD^+^-GDH activity by altering the affinity of the enzyme for its substrate [29]. This binding, however, is prevented by the phosphorylation of GarA [29] by PknG. The conditions under which PknG is stimulated to phosphorylate or dephosphorylate GarA has not yet been investigated and it is not clear how the relationship between GarA, NAD^+^-GDH and PknG may impact nitrogen metabolism in the mycobacteria.

The physiological roles as well as the regulation of the major effectors of nitrogen metabolism (GS and GDH) in *M. smegmatis *remains unclear. As the adaptive mechanisms of the mycobacteria to limited nitrogen availability remain vague, an investigation into the changes in activity and transcription of both glutamine synthetase and the glutamate dehydrogenase enzymes under various conditions of ammonium availability in *M. smegmatis*, as a model for the mycobacteria, has been undertaken. 

## Results and Discussion

### GDH specific activity in response to ammonium limitation and excess

To investigate the effect of nitrogen availability on GDH activity, *M. smegmatis *was cultured in minimal medium containing a limited amount of ammonium (3 mM (NH_4_)_2_SO_4_). The specific activity of both the aminating and deaminating reactions catalysed by NAD^+^- and NADP^+^-GDH (see Reaction 2) was determined from *M. smegmatis *whole cell lysates sampled at 0; 0.5; 2 and 4 hour intervals. The effect of an ammonium pulse (60 mM (NH_4_)_2_SO_4_) on GDH activity was determined after 0.5 and 1 hours exposure to those conditions.

**Figure 2:**
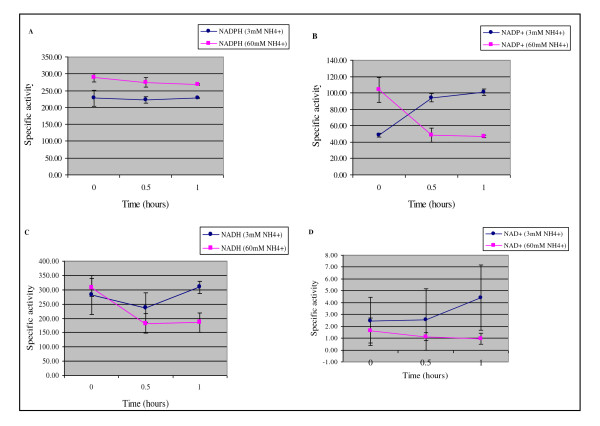
**Specific activities of the (A) NADP^+^-specific forward reaction in which NADPH was added as co-factor, (B) NADP^+^-specific reverse reaction in which NADP^+ ^was utilised as co-factor, (C) NAD^+^-specific forward reaction with NADH as co-factor and (D) NAD^+^-specific reverse reaction in which NAD^+ ^was utilised as co-factor**. Each enzyme reaction was assayed under conditions of nitrogen limitation (3 mM (NH_4_)_2_SO_4_) and in response to an ammonium pulse (60 mM (NH_4_)_2_SO_4_). One unit of enzyme activity was defined as the oxidation/reduction of 1 nmole co-factor per minute per milligram ofprotein. The mean specific activity with standard deviations is included.

The NADP^+^-GDH forward or aminating reaction activity in *M. smegmatis *did not change appreciably in response to ammonium availability as can be seen by the absence of any significant change in activity between 0 hr and 0.5 or 1 hr nitrogen starvation (Figure 2A, ●). This was also true for *M. smegmatis *exposed to an ammonium pulse (Figure 2A, ■). It would appear as though the NADP^+^-GDH aminating reaction activity of *M. smegmatis *exposed to nitrogen limitation remained greater than that of *M. smegmatis *exposed to ammonium excess conditions (Figure 2A). This, however, could be misleading as, at certain time points, the bacteria were exposed to similar conditions of nitrogen availability in each experiment. For example, *M. smegmatis *incubated for 1 hr in media containing 60 mM NH_4_^+ ^at time point 0 hr before being starved of nitrogen (Figure 2A, ●) was the same as after 1 hr exposure to ammonium excess conditions (Figure 2A, ■). The activity of the NADP^+^-GDH reaction is expected to be relatively similar under homologous conditions, thus the disparity observed may be due to slight experimental differences in the amount of starting material, assay conditions or absorbance readings measured during the activity assays. Our results also show that NADP^+^-GDH aminating reaction activity did not change significantly in response to prolonged exposure to nitrogen limitation (Table 1). This lack of change in *M. smegmatis *NADP^+^-GDH reaction activity is in contrast to a recent study in which NADP^+^-GDH animating activity was found to increase significantly in response to nitrogen starvation in a related Actinomycete, *Corynebacterium glutamicum *[36]. In other bacterial species, NADP^+^-GDH forward reaction activity has been shown to be down-regulated in response to nitrogen excess [37,38] or not regulated at all [39].

**Table 1:** Specific activities of the both the aminating and deaminating reactions for NADP- and NAD-glutamate dehydrogenase enzymes in response to nitrogen starvation conditions (3 mM (NH_4_)_2_SO_4_).

	Specific Activity (U)							
	
Time (hours)		p-value*		p-value*		p-value*		p-value*
	**NADPH**		**NADP**^+^		**NADH**		**NAD**^+^	
0	227 ± 24		49 ± 2		281 ± 67		0.02 ± 3	
0.5	222 ± 9	0.76	94 ± 5	**0.01**	264 ± 51	**0.01**	2.57 ± 3	0.99
1	229 ± 2	0.71	101 ± 4	0.69	309 ± 21	**0.00**	4 ± 3	0.91
2	231 ± 10	0.91	103 ± 9	0.80	309 ± 41	0.98	2 ± 2	0.91
4	209 ± 11	0.20	102 ± 25	0.85	356 ± 19	**0.01**	0.16 ± 3	1.00

*P-values less than 0.05 (in bold print) were regarded as statistically significant changes in specific activity from the previous time point.

Upon analysis of the NADP^+^-GDH reverse or deaminating reaction activity, our results showed that there was a significant change in activity in response to nitrogen availability in *M. smegmatis *(Figure 1B) thereby suggesting NADP^+^-GDH is indeed regulated in response to varying nitrogen concentration conditions. When exposed to ammonium starvation conditions, there was a 2 fold increase (Figure 2B, ● and Table 1, p = 0.01) in NADP^+^-GDH deaminating reaction activity (i.e. glutamate catabolism), which remained constant throughout an extended period of nitrogen starvation (Table 1). The converse effect was observed under conditions of nitrogen excess, namely a rapid, approximately 2 fold decrease in reaction activity (Figure 2B, ■). Since NADP^+^-GDH performs a reversible reaction, it is interesting to note that a change only in the deaminating reaction activity in response to nitrogen availability was detected. The functional significance of the observed change in glutamate deamination is unclear. It may be that the modulation of glutamate catabolism serves to maintain the intracellular glutamate/2-oxoglutarate ratios which is known to co-ordinate a number of cellular activities such as growth rate modulation [40]; maintenance of intracellular potassium pools [41] and protection from high osmotic environments [42].

There are two possible NAD^+^-GDH enzymes encoded by the *M. smegmatis *genome. The highly NAD^+ ^specific GDH encoded by *msmeg_4699 *was isolated and characterised by O'Hare *et al*. [29] which showed great similarity to the novel class of large GDH enzymes known as the L_180 class [18]. The second putative NAD^+^-GDH is encoded by *msmeg_6272 *and has an approximate subunit size of 118 kDa [43]. This enzyme may fall into the 115 kDa class of large GDH's, however the presence of a functional protein is yet to be shown. Under our experimental conditions, the total NAD^+^-GDH deaminating reaction activity was very low and did not notably alter in response to changing ammonium concentrations (Figure 2D) nor to prolonged ammonium starvation conditions (Table 1). This observation may be attributable to the very low glutamate affinity of the L_180 class of NAD^+^-GDH (MSMEG_4699) [29]. In contrast, the NAD^+^-GDH aminating reaction activity was much higher and was significantly changed by ammonium availability (Figure 2C). During nitrogen starvation, the total NAD^+^-GDH aminating activity tended to increase (a 14% increase between 0.5 and 1 hrs, p = 0.00, Table 1) and remained elevated but relatively constant throughout the ammonium starvation time course study (Table 1), presumably in order to assist nitrogen assimilation under these conditions. In response to an ammonium pulse, the total NAD^+^-GDH aminating activity was reduced almost 2 fold (p = 0.00, data not shown; Figure 2C, ■). This decrease in activity may be due to the presence of a constitutively active NADP^+^-GDH which could adequately assimilate nitrogen under these conditions. In *M. smegmatis*, it would appear that at least one of the possible NAD^+^-GDH enzymes plays a largely anabolic or aminating role, which is in contrast with the opinion that NAD^+^-GDH enzymes are normally involved in glutamate catabolism [12,13]. In addition, it would appear that at least one of the NAD^+^-GDH enzymes present in *M. smegmatis *is regulated in response to nitrogen availability. It may be that the regulation of NAD^+^-GDH activity in response to nitrogen availability may be due to the interaction of non-phosphorylated GarA with the enzyme under conditions of nitrogen excess and this interaction may be abolished by pknG mediated phosphorylation of GarA under conditions of nitrogen starvation.

### Glutamine synthetase specific activity in response to ammonium limitation and excess

The activity of the high ammonium affinity GS enzyme was assessed using the γ-glutamyl transferase assay [44]. Upon exposure to nitrogen limitation, *M. smegmatis *GS activity increased significantly (p = 0.01) within 0.5 hrs and continued to increase significantly after 1 hr of nitrogen starvation (Table 2) to reach a final activity at 4 hrs that was approximately 2.5 fold greater than at zero hours (Table 2). When an ammonium pulse was applied to nitrogen starved cells, GS activity decreased significantly (0.66 fold reduction, p = 0.00, Table 2) within 1 hr of exposure to nitrogen excess. Our results are in accordance with studies done in a variety of bacteria, including *M. tuberculosis*, which have shown that GS activity is up-regulated (approximately 3.7 fold in *M. tuberculosis *[45]) in response to nitrogen limitation and conversely regulated in response to nitrogen excess [45,46]. In *M. tuberculosis*, this regulation is achieved by post-translational adenylylation of GS [3,45], and transcriptional control [47]. These results indicate that, under our experimental conditions, *M. smegmatis *did sense 3 mM (NH_4_)_2_SO_4 _as a nitrogen starvation condition since GS activity was up-regulated, most likely in order to scavenge ammonium from the environment. In addition, 60 mM (NH_4_)_2_SO_4 _was perceived as a condition of nitrogen sufficiency, as GS activity was down-regulated in order to prevent a futile energy depleting cycle.

**Table 2:** Glutamine synthetase specific activities determined by the γ-glutamyl transferase assay when *M. smegmatis *was exposed to conditions of nitrogen limitation (3 mM (NH_4_)_2_SO_4_) and nitrogen excess (60 mM (NH_4_)_2_SO_4_).

(NH_4_)_2_SO_4 _Concentration (mM)	Time (hours)	Specific activity (U)	p-value*
**3 mM**	**0**	45 ± 17	
	
	**0.5**	57 ± 12	**0.01**
	
	**1**	63 ± 12	0.27
	
	**2**	78 ± 16	**0.00**
	
	**4**	103 ± 17	**0.00**

**60 mM**	**0**	76 ± 2	
	
	**0.5**	50 ± 1	**0.00**
	
	**1**	47 ± 5	0.08

* The p-values given show the statistical significance of the change in GS specific activity between time points. p < 0.05 (in bold) was regarded as a statistically significant change in specific activity from the previous time point.

### Relative quantification of gene transcription

The response to nitrogen availability at the mRNA level of genes encoding for GS (*glnA1*), NADP^+^-GDH (*msmeg_5442*) and the L_180 NAD^+^-GDH (*msmeg_4699*) was assessed by semi-quantitative Real-Time PCR [48]. The relative change in gene expression was calculated as a ratio of target gene transcription versus the transcription of *sigA*, as an internal control.

A significant up-regulation (factor of 2 ± 0.5, p = 0.001, Table 3) of *glnA1 *gene transcription was observed within 0.5 hrs exposure to nitrogen starvation and continued to increase significantly thereafter (Table 3). This was an expected result as similar increases have been reported in *M. smegmatis *[49]. Within the first hour, the increase in gene transcription was relatively low which indicated that the requirement for the synthesis for additional GS protein was not very high. It has previously been reported that a surprisingly large quantity of GS is produced by *M. tuberculosis *and is exported to the extracellular milieu [23]. Although *M. smegmatis *does not export GS [23], it may be that, similar to *M. tuberculosis*, a large intracellular pool of GS is present whose activity could be up-regulated via de-adenylylation by GlnE [45] and thereby satisfy the need for ammonium assimilation under these conditions. After 2 hrs exposure to nitrogen starvation, there was a profound increase in *glnA1 *transcription (67 ± 38, Table 3) which may reflect a heightened state of intracellular nitrogen starvation and thus the requirement for increased levels of GS enzyme in order to efficiently assimilate ammonium under these conditions. The relatively constant increase in GS activity under the same conditions (Table 2) was most likely due a combination of an increase in *glnA1 *transcription and very strict control of GS activity by the adenylyltransferase, GlnE, in order to balance ammonium assimilation; energy expenditure and the intracellular glutamate/glutamine ratios. When an ammonium pulse was applied to *M. smegmatis *that had been starved of nitrogen, a down-regulation in transcription was observed, however, it was not found to be statistically significant (data not shown). There was, however, a rapid and significant decrease in GS specific activity when the bacteria were exposed to an ammonium pulse (Table 2) which suggests that post-translational modification via GlnE is responsible for the swift response in GS activity to changing ammonium concentrations.

**Table 3:** Relative quantification^a ^of the expression of GS (*glnA1*), NADP-GDH (*msmeg_5442*) and L_180 NAD-GDH (*msmeg_4699*) when *M. smegmatis *was exposed to prolonged periods of nitrogen limitation.

**Time (hours)**	**Gene**					
	
	***glnA1***	**P-value**	***MSMEG_4699***	***P-value***	***MSMEG_5442***	**P-value**
	
**0.5**	2 ± 0.5	0.001	0.5 ± 0.1	0.001	0.5 ± 0.1	0.001
**1**	3 ± 0.6	0.001	0.6 ± 0.05	0.001	0.5 ± 0.08	0.001
**2**	67 ± 38	0.001	13 ± 4	0.001	2 ± 0.9	0.901
**4**	58 ± 43	0.001	18 ± 15	0.001	3 ± 3	0.272

^a ^The relative change in gene expression when *M. smegmatis *was exposed to nitrogen starvation was compared to gene expression after *M. smegmatis *exposed to 60 mM (NH_4_)_2_SO_4 _for 1 hours (time point zero). *SigA *was used as the internal reference gene. Values >1 reflect an up-regulation of gene expression whereas values <1 represent a down-regulation of expression in relation to the non-regulated internal reference, *sigA*.

* statistically significant gene regulation (p < 0.05)

**Figure 3:**
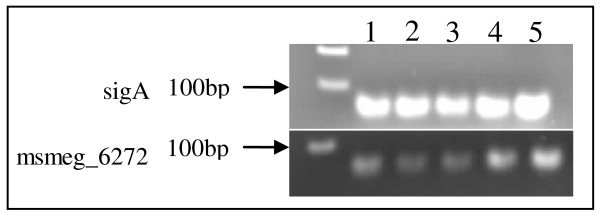
**Reverse transcriptase PCR of *msmeg_6272 *cultured under conditions of nitrogen starvation (3 mM (NH_4_)_2_SO_4_) for four hours**. Lane (1) 0 hr at which point *M. smegmatis *was exposed to nitrogen excess (60 mM (NH_4_)_2_SO_4_) for 1 hr (2) 0.5 hr nitrogen starvation; (3) 1 hr nitrogen starvation (4) 2 hrs nitrogen starvation and (5) 4 hrs nitrogen starvation. *SigA *was amplified as an unregulated internal control.

Within the first hour of nitrogen limitation, the transcription of both *msmeg_5442 *and *msmeg_4699 *was statistically significantly down-regulated by a relative factor of 2.00 (calculated by 1/expression ratio). The expression of *msmeg_5442 *did not alter significantly thereafter (Table 3). The down-regulation of NADP^+^-GDH (*msmeg_5442*) observed in *M. smegmatis *is similar to the pattern of expression of the homologous gene (*SCO4683*) in a related Actinomycete, *Streptomyces coelicolor*, under analogous conditions [50]. The L_180 class of NAD^+^-GDH enzymes identified to date have been well characterised, however, the expression of the genes encoding these enzymes has not yet been investigated in any depth. Under our experimental conditions, the L_180 NAD^+^-GDH in *M. smegmatis *(*msmeg_4699*) was down-regulated within the first hour of nitrogen starvation (Table 3). However, after 2 hrs exposure to nitrogen starvation conditions, there was a statistically significant increase in *msmeg_4699 *transcription (factor of 13 ± 4, p = 0.001, Table 3). The expression of the putative NAD^+^-GDH gene, encoded by *msmeg_6272*, was also analysed but by reverse transcriptase PCR. The PCR products were separated on a 1% agarose gel which were quantified using densitometric analysis of the gel image [51]. An *msmeg_6272 *mRNA species was detected (Figure 3) which indicated that the gene was transcribed under our experimental conditions. In addition, from visual inspection of the gel image (Figure 3), *msmeg_6272 *appeared to be regulated in response to nitrogen availability. Upon densitometric analysis, it was found that after an initial 2 fold decrease in gene expression (Table 4) in response to nitrogen starvation, gene transcription appeared to be up-regulated after 2 hrs (approximately 2 fold, Table 4) exposure to these conditions.

**Table 4:** Relative quantification of *msmeg_6272 *by reverse transcriptase PCR under conditions of nitrogen limitation (3 mM (NH_4_)_2_SO_4_) and excess (60 mM (NH_4_)_2_SO_4_).

Culture condition	Time (hrs)	Fold Increase (+) or Decrease (-) in expression
**3 mM (NH_4_)_2_SO_4_**	0.5	- -
	
	1	no change
	
	2	+ +
	
	4	no change

**60 mM (NH_4_)_2_SO_4_**	0.5	no change

Transcriptional control of nitrogen-related genes in *S. coelicolor *is co-ordinated by an OmpR-type regulator, GlnR, which can act both as an activator and repressor of transcription [50,52]. A GlnR-type regulator has been identified in *M. smegmatis *and has been shown to regulate a number of nitrogen-related genes in this organism[49]. Amon *et al*. [49] were able to elucidate a GlnR consensus DNA binding sequence, however, this binding sequence could not be identified upstream of *msmeg_5442 *[49] and has not been investigated with regards to *msmeg_4699 *or *msmeg_6272*. The *M. smegmatis *genome also encodes for a putative TetR-type transcriptional repressor, AmtR, which is responsible for the regulation of a number of genes involved in nitrogen metabolism in *C. glutamicum *[53]. The gene encoding for NADP^+^-GDH in *C. glutamicum *is up-regulated in response to nitrogen starvation, however, it was found that the transcription of this gene is highly variable and is controlled by a variety of regulators [10] including AmtR. It is possible that either of these regulators may be responsible for the regulation of *msmeg_5442; msmeg_6272 *and *msmeg_4699 *transcription in *M. smegmatis*, however, this remains to be investigated.

The observed transcriptional regulation of the gene encoding for NADP^+^-GDH (*msmeg_5442*) did not directly correlate with observations made at the level of GDH specific activity. An initial down-regulation of *msmeg_5442 *gene transcription was seen under conditions of nitrogen starvation (Table 3), yet NADP^+^-GDH reaction activity increased (Figure 2B). This result suggests that an additional regulatory mechanism may play a role in the control of total NADP^+^-GDH enzyme activity. A slightly different trend was observed for NAD^+^-GDH under conditions of nitrogen starvation. The expression of *msmeg_6272 *and *msmeg_4699 *was repressed within the first hour of nitrogen starvation (Table 4) which was reflected by an initial decrease in NAD^+^-GDH specific activity. However, between 0.5 hr and 1 hr nitrogen starvation, there was a significant increase in NAD^+^-GDH specific activity in the absence of an increase in transcription of either *msmeg_4699 *or *msmeg_6272 *(Table 3 and 4). After 2 hrs exposure to nitrogen starvation conditions, the expression of *msmeg_4699 *and *msmeg_6272 *increased significantly (by a factor of approximately 5 and 2, respectively, Table 3) which, once again, was mirrored by an increase in specific activity of NAD^+^-GDH by approximately 50 U (Table 1). These observations suggest that NAD^+^-GDH activity may be regulated by both transcriptional control and an additional regulatory mechanism such as post-translational modification.

## Conclusion

The production of glutamate and glutamine is critically important in all bacteria for the synthesis of essential cellular components. Glutamate can be produced by either GOGAT or GDH and glutamine is produced by glutamine synthetase via the GS/GOGAT cycle. The large energy cost associated with the production of glutamate and glutamine by the GS/GOGAT system can be bypassed by the functioning of the GDH pathway (if present) under conditions of nitrogen excess. Conversely, under nitrogen limiting conditions, the GS/GOGAT cycle becomes the major nitrogen assimilatory route (for review see [54]). Our analysis of *M. smegmatis *GS found that both enzyme specific activity and *glnA1 *transcription were regulated in response to nitrogen availability. GS specific activity was rapidly down-regulated under excess ammonium concentrations and conversely regulated when starved of ammonium. This rapid change in activity, in the absence of initial significant transcriptional regulation, could be attributed to post-translational control by GlnE. The large increase in *glnA1 *transcription after a prolonged period of nitrogen starvation (2 to 4 hrs ammonium starvation) could, together with post-translational regulation, be responsible for further increases in GS activity under those conditions. GS appeared to play a greater assimilatory role under conditions of nitrogen limitation than under conditions of nitrogen excess which is similar to observations made in other bacteria [46].

Under our experimental conditions, we observed that NADP^+^-GDH aminating activity did not alter significantly in response to nitrogen availability, in contrast to results obtained in related Actinomycetes such as *S. coelicolor *[55] or *C. glutamicum *[36]. It appeared as though NADP^+^-GDH in *M. smegmatis *had a constitutive ammonium assimilatory function under our experimental conditions. It was found, however, that the de-aminating activity of NADP^+^-GDH did change in response to nitrogen availability which suggests that the activity of NADP^+^-GDH in *M. smegmatis *is regulated in a manner different to other Actinomycetes. It may be that an increase in glutamate catabolism under these conditions could produce free ammonia required for essential glutamine production by GS. The high levels of NAD^+^-GDH aminating activity observed under all conditions of ammonium availability in *M. smegmatis *was unexpected as NAD^+^-GDH enzymes are presumed to be largely involved in glutamate catabolism. In addition, NAD^+^-GDH animating activity appeared to change in response to nitrogen availability which could indicate an important role in ammonium assimilation. In the absence of an initial upregulation of NAD^+^-GDH gene transcription under conditions of ammonium starvation, the observed increase in NAD^+^-GDH aminating activity might possibly be attributed to other control mechanisms, such as the GarA-pknG regulatory system. This type of regulation may also account for the observed decrease in NAD^+^-GDH aminating activity upon exposure to an ammonium pulse. Transcription of *msmeg_4699 *and *msmeg_6272 *increased after prolonged exposure to nitrogen starvation (2 to 4 hrs ammonium starvation), which similarly to GS, could contribute to the maintenance of elevated levels of activity under those conditions. An inherent limitation of this study is that cell free extracts were used in enzyme activity assays which may possibly contain enzymes/proteins other than the glutamate dehydrogenases that could utilize NAD(P)H as co-factors and therefore confound GDH assay results. However, since whole cell lysates have been utilized successfully in previous studies [10,37,56], the possibility that the observed changes in enzyme activity are true physiological responses to nitrogen availability should not be disregarded.

From our results, it would appear that there are differences in the roles that the various GDH enzymes play in *M. smegmatis *and in other related organisms. There are also differences between the mycobacteria. The slow growing pathogenic mycobacteria such as *M. tuberculosis *and *M. bovis *do not appear to have an NADP^+^-GDH, however both genomes do encode for an NAD^+^-GDH which share a 81% and 82% amino acid identity with MSMEG_4699 respectively. The results obtained from our study imply that NAD^+^-GDH may play a previously unpredicted and potentially important nitrogen assimilatory role in these pathogenic species. Since NAD^+^-GDH enzymes are able to assimilate nitrogen with a much lower associated energy cost than the GS/GOGAT system, it is possible that these enzymes may facilitate bacterial survival under conditions where energy preservation is vital, such as during latency. An investigation into the physiological roles of NAD^+^-GDH enzyme in *M. bovis *is currently underway.

## Methods

### Bacterial strains and culture methods

*Mycobacterium smegmatis MC155*^2 ^was routinely cultured in 7H9 medium (Difco) supplemented with 10% Oleic acid-Albumin-Dextrose-Catalase enrichment (OADC; Middlebrook) until an OD_600 _of approximately 0.8. The bacteria were transferred to Kirchner's minimal medium [57] in which asparagine was replaced with ammonium sulphate ((NH_4_)_2_SO_4_) as the sole nitrogen source. It has previously been shown that an increase in NH_4_^+ ^concentration from 3.8 mM to 38 mM caused a 10-fold reduction in *M. tuberculosis *activity [23]. The observed response of GS activity to the change in NH_4_^+ ^concentration is indicative that bacteria exposed to 3.8 mM NH_4_^+ ^were starved of nitrogen. In addition to a change in activity, a response in the level of GS transcription was also observed [47]. An (NH_4_)_2_SO_4 _concentration of 3 mM was thus used to induce nitrogen starvation in *M. smegmatis *whereas Kirchner's medium containing 60 mM (NH_4_)_2_SO_4 _was considered as nitrogen sufficiency or excess. *M. smegmatis *liquid cultures were maintained at 37°C with shaking.

### Preparation of crude protein extract

*M. smegmatis *was harvested by centrifugation and resuspended in 1 ml of Tris-HCl (pH 8) or phosphate buffer (Na_2_H_2_PO_4_/K_2_HPO_4_; pH 7.0). The cells were disrupted by ribolysing at maximum speed for 20 sec (Fastprep FP120, Bio101 Savant) and immediately placed on ice for 1 min thereafter. This ribolysing procedure was repeated 3 to 4 times with intermittent cooling on ice. The sample was centrifuged at 4°C in a benchtop centrifuge (Mikro 200, Hettich Zentrifugen) to remove insoluble material and the total protein concentration was determined using the Bradford assay (Bio-Rad, Germany) according to the manufacturer's instructions.

### Enzyme assays

#### Glutamate dehydrogenase activity assays

##### i) NADPH-specific Glutamate dehydrogenase

NADPH-GDH activity was assayed essentially as described by Sarada *et al*. [28]. The NADPH-GDH forward reaction (reductive aminating activity) was assayed by preparation of a 1 ml reaction system containing 100 mM Tris HCl (pH 8.0), 100 mM NH_4_Cl; 10 mM α-ketoglutarate and 0.1 mM NADPH. The NADPH-GDH reverse reaction (oxidative deaminating activity) assay preparation consisted of 100 mM Tris-HCl (pH 9.0); 200 mM glutamate and 0.1 mM NADP^+^. The reactions were initiated by the addition of 10 μg *M. smegmatis *crude protein extract.

##### ii) NADH-specific GDH

The activity of both the forward and reverse NADH-GDH reactions were assayed using a combination of methods from Loyola-Vargas *et al*. [56] and Miñambres *et al*.[18]. The 1 ml NADH-GDH forward reaction (reductive amination) assay consisted of 100 mM Phosphate buffer (HK_2_PO_4_/H_2_NaPO_4_; pH 7.0); 100 mM NH_4_Cl; 10 mM α-ketoglutarate and 0.16 mM NADH. The 1 mL reverse reaction assay (oxidative deamination) was prepared by adding 100 mM Phosphate buffer (pH 7.0); 100 mM L-glutamate; and 2 mM NAD^+^. The assay reactions were initiated by the addition of 10 μg *M. smegmatis *crude protein extract.

The forward or aminating reactions were assayed by measuring the oxidation of NADPH or NADH spectrophotometrically at 340 nm. The reverse or deaminating reactions were assayed by measuring the reduction of NADP^+ ^or NAD^+ ^at 340 nm. Specific enzyme activities were calculated using the NAD(P)H extinction co-efficient of 6.22 cm^2^/μmole. One unit of enzyme activity was defined as 1 nmole of coenzyme (NAD(P)H) oxidized or reduced per minute, per milligram protein added. A two-way ANOVA using a mixed model with the correct nested terms was used to analyse the data.

#### Glutamine synthetase activity assay

Total GS activity was assayed using the γ-glutamyl-transferase assay as described elsewhere [58]. Briefly, total GS activity was assayed in the presence of 0.3 mM Mn^2+ ^as the activity of both adenylylated and de-adenylylated forms of GS are measured under these conditions. The reaction was initiated by the addition of 10 μg *M. smegmatis *crude protein extract and allowed to proceed for 30 min at 37°C. The reaction was halted by the addition of a stop mix (1 M FeCl_3_.6H_2_O, 0.2 M Trichloroacetic acid and 7.1% v/v HCl) and the samples were briefly centrifuged in order to remove any precipitate that may have formed. The production of γ-glutamylhydroxamate was determined by measuring the absorbance at 540 nm. One unit of enzyme activity was defined as the amount of enzyme producing 1 μmole γ-glutamylhydroxamate/min/mg protein in the transfer reaction. A technical replicate of each enzyme assay was measured and each experiment was repeated at least three times. A two-way ANOVA using a mixed model with the correct nested terms was used to analyse the data.

### RNA preparation

*M. smegmatis *cells were collected by centrifugation (Eppendorf Centrifuge 5810R) and resuspended in 1 ml Trizol (Invitrogen). The cell suspension was ribolysed (Fastprep FP120, Bio101 Savant) in a 2.0 ml screw cap microtube (Quality Scientific Plastics) containing 0.5 mm glass beads at a maximum speed setting of 6.0 for 20 seconds. The tubes were immediately placed on ice for 1 minute to dissipate the heat caused by friction during the ribolyzing process. This homogenisation step was repeated 3-4 times and the cooled homogenate was incubated at room temperature for 5 minutes to allow dissociation of nucleoprotein complexes. A total of 250 μl chloroform was added to the mixture which was rapidly inverted for the first 20 seconds, and then periodically thereafter for a further 5 minutes at room temperature. The samples were centrifuged at 18630 × g (4°C) for 10 min and the aqueous phase removed. Two volumes of ice-cold 100% ethanol was added to the supernatant, mixed by inversion and incubated at -20°C overnight to allow nucleic acid precipitation. RNA was collected by centrifugation at 18630 × g (4°C), washed with 70% ethanol and resuspended in water. Any contaminating DNA was removed by DNase digestion (Turbo-DNase, Ambion) according to the manufacturer's instructions. Quality and quantification of total bacterial mRNA extracted was assessed using the Experion system (Experion RNA Standard Sense Kit, Bio-Rad).

Complementary DNA was synthesised from 1 μg total RNA using the Transcriptor First Strand cDNA Synthesis Kit (Roche) and random hexamer primers (supplied) according to manufacturer's instructions.

### Real-time and reverse-transcriptase PCR

Real-Time PCR reactions were performed in the LightCycler version 1.5 (Roche Diagnostics) using either the LightCycler Master^Plus ^SYBR Green (Roche) or the Master SYBR Green kit (Roche). PCR master mixes (SYBR Green dye and FastStart Taq DNA polymerase were supplied) were prepared according to the manufacturer's instructions. A four step experimental protocol was used: (i) activation (95°C for 15 min) (ii) amplification step repeated for 45 cycles (95°C for 10 sec; primer-specific Tm for 10 sec, 72°C for 10 sec with a single fluorescence measurement) (iii) melting curve analysis (65°C-95°C with a heating rate of 0.1°C per second and a continuous fluorescence measurement) (iv) cooling step down to 40°C (see Table 1 for annealing temperatures). Refer to Table 5 for a complete list of primer sequences used to analyse the genes of interest. RNA template and no-template controls were included to determine DNA contamination of RNA samples or PCR reactions. All PCR reactions as well as all biological experiments were done in triplicate. Relative quantification of gene expression was done using the REST-384 Version 1 software with PCR efficiency correction for individual real-time PCR transcripts [48]. *SigA *was used as the internal standard to normalise target gene expression levels in each RNA sample [59] as it has been shown that *sigA *expression remains constant under various growth and stress conditions [60].

**Table 5:** Primer sequences used for the relative quantification of glutamine synthetase and glutamate dehydrogenase genes.*

Gene	Sense Primer (5'-3')	Antisense Primer (5'-3')	Product size (bp)	Annealing Temperature (°C)
*glnA1*	ATGTGCTGCTGTTCAAGT	TGAAGGTGACGGTCTTGC	66	55
*sigA*	GACTCGGTTCGCGCCTA	CCTCTTCTTCGGCGTTG	64	55
*msmeg_6272*	TGATCCGCCACATCCTG	GATGTAGGTGCCGATGC	65	56.5
*msmeg_5442*	AGATCATGCGGTTCTGTC	GTGTATTCACCGATGTGCC	61	55
*msmeg_4699*	GTGAGGACTTCCGCACC	CCGCTTGACGACGAATC	104	55

*The product size and annealing temperatures are also given. *sigA *was used as an internal control or housekeeping gene. Reverse transcriptase PCR reactions were carried out in the GeneAmp PCR System 9700

Reverse transcriptase PCR reactions were carried out in the GeneAmp PCR System 9700 (Applied Biosystems) using HotStar Taq DNA Polymerase (Qiagen) according to manufacturer's instructions. A 1/100 dilution of the cDNA was made and equal volumes (2 μl) were used in each PCR reaction. The PCR protocol was initiated by an activation step of 15 min at 95°C. This was followed by 35 cycles of: denaturation (95°C for 30 sec), primer annealing (Tm specific for 30 sec) and elongation (72°C for 30 sec). A final DNA polymerisation step at 72°C for 10 min followed by cooling to 4°C was included. Densitometric analysis of gel images (Un-Scan-It gel Automated Digitizing System, Version 5.1) was used to quantify gene expression [51].

## Authors' contributions

CJH conceived of the study, performed the enzyme assays, transcriptional studies and drafted the manuscript. DH was involved in the study design and participated in glutamine synthetase assays. MK did all statistical analyses on acquired data. IW participated in the design of the study, contributed to the analysis of the data and revision of the manuscript. PvH was involved in the interpretation of the data and critical revision of the manuscript. All authors have read the manuscript and approved the final product.
